# *Bartonella* tracing in wild rodents in northwestern Mexico

**DOI:** 10.1017/S0950268825000238

**Published:** 2025-02-28

**Authors:** Adriana M. Fernández-González, Andrés M. López-Pérez, Angel Herrera-Mares, Andrea Chaves, Fabiola Ramírez-Corona, Gerardo Suzán

**Affiliations:** 1 Departamento de Etología, Fauna Silvestre y Animales de Laboratorio, Facultad de Medicina Veterinaria y Zootecnia, Universidad Nacional Autónoma de México, Ciudad de México, México; 2 Red de Biología y Conservación de Vertebrados, Instituto de Ecología A.C., Veracruz, México; 3 Centro Nacional de Innovaciones Biotecnológicas (CENIBiot), CeNAT-CONARE, San José, Costa Rica; 4 Escuela de Biología, Universidad de Costa Rica, San José, Costa Rica; 5 Taller de Sistemática y Biogeografía, Departamento de Biología Evolutiva, Facultad de Ciencias, Universidad Nacional Autónoma de México, Ciudad de México, México

**Keywords:** *Bartonella*, rodent, Mexico, Chihuahua, Baja California

## Abstract

*Bartonella* is a widely distributed Gram-negative bacterium that includes species that are capable of causing illness in humans. Rodents represent one of the main reservoirs of zoonotic pathogens, and monitoring their populations can provide valuable insights into human health. We conducted a surveillance study of rodents from two north-western states of Mexico (Baja California and Chihuahua) to investigate the prevalence and genetic diversity of *Bartonella* by polymerase chain reaction (PCR) amplification and sequencing of the citrate synthase (*gltA*) gene. A total of 586 rodents belonging to 28 species were captured, and 408 were tested for *Bartonella* spp. The overall *Bartonella* spp. prevalence was 39.71%. The prevalence found in Chihuahua was higher (42.80%) than in Baja California (32.52%), and rodents such as *Neotoma albigula*, *Neotoma mexicana*, *Peromyscus boylii*, and *Chaetodipus baileyi* had the highest prevalence. The *gltA* sequences revealed seven genetic variants, some of which were obtained from *Peromyscus* and *Dipodomys* rodents and were associated with *Bartonella* species of human health concern, such as *B. grahamii* and *B. vinsonii* subsp. *arupensis.* In addition, a sequence obtained from a *Peromyscus maniculatus* was clustered with *Candidatus* Bartonella rudakovii, a previously unreported association. This study provides valuable data and new insight into the *Bartonella*-hosts interactions in rodent species in north-western Mexico.

## Introduction

*Bartonella* is a genus of Gram-negative bacteria with an affinity for erythrocytes and endothelial cells of various hosts, including agents of emerging or re-emerging infectious diseases [1]. These bacteria are widely distributed around the world and parasitize a wide range of mammals, including rodents and humans [2, 3]. Currently, 45 species of *Bartonella* are recognized, of which at least ten are associated with human illness [4]. The extensive range of hosts that are parasitized by this bacterium and its remarkable richness can be attributed in part, to the transmission route, which is through numerous ectoparasitic vectors (fleas, lice, sand flies, among others) [5]. Furthermore, the high degree of adaptation exhibited by *Bartonella* to its reservoir hosts facilitates prolonged intraerythrocytic bacteremia and persistent infection of endothelial cells, thereby enabling these reservoir hosts to serve as foci of infection [1, 6].

The order Rodentia is the most abundant and diverse of mammals and has a great richness of *Bartonella* species [7, 8]. Ectoparasitic vectors, such as fleas, play an important role in the *Bartonella*-rodent cycle. Fleas are capable of moving between hosts and feeding on them, which provides the opportunity for *Bartonella* spp. to infect different hosts and fleas. This may explain the great richness and diversity of *Bartonella* found in rodents and their fleas [7, 9]. In addition to this, the order Rodentia shows a strong association between specific *Bartonella* species and certain rodent hosts. For example, specificity has been reported for *Bartonella washoensis* in sciurid rodents [10] and between *Bartonella vinsonii* subsp. *arupensis* and rodents of the genus *Peromyscus* in the United States and Mexico [11, 12]. Specificity has also been observed between *Bartonella* and fleas. For example, fleas of the species *Oropsylla hirsuta* which are specific to Sciuridae such as *Cynomys ludovicianus* are also infected by *B. washoensis*, and fleas of the genus *Orchopeas* which parasitize rodents of the genus *Peromyscus* are also infected by *B. vinsonii* subsp. *arupensis* [9, 13]. However, studies indicate that infection by more than one species or subspecies of *Bartonella* is possible in rodents and fleas. A longitudinal study in Georgia found multiple genogroups isolated from individual blood samples obtained from *Sigmodon hispidus* rodents, which occurred in 21% of the blood samples obtained; and another study conducted in Israel with *Xenopsylla ramesis* fleas and wild rodents indicates a co-infection of two different strains of *Bartonella* in *Meriones crassus* and *Gerbillus nanus* rodents [14, 15]. Regarding fleas, a study on wild mammals and their fleas demonstrated a co-infection with two different strains of *Bartonella* in fleas *Aetheca wagneri* and *Orchopeas leucopus*, collected from *Peromyscus maniculatus* rodents [16]. Therefore, in the *Bartonella*-rodent-flea cycle, some specificity of the bacteria with certain species of fleas and rodents can be observed; however, there is also the possibility of finding co-infection of different species and subspecies of *Bartonella* in rodents and their fleas which could give the opportunity for genetic recombination and thus diversification of this bacterium [17].

In North America, a high prevalence of *Bartonella* has been reported in several rodents. In Kansas, a prevalence of 90.4% was reported in *Onychomys leucogaster* rodents; in Colorado, the prevalence in *P. maniculatus* was 82.4% [11, 18]; in New Mexico, the prevalence in *Neotoma* rodents was 64% [19]; and in northern Mexico, the prevalence of *Bartonella* infection was found to be significantly higher in *Dipodomys merriami* (57%), *Dipodomys spectabilis* (51%), *Onychomys arenicola* (80%), *O. leucogaster* (83%), *Peromyscus leucopus* (50%), and *P. maniculatus* rodents (50%) [12].

The infection caused in humans by different species of *Bartonella* is called bartonellosis and includes several diseases such as cat scratch disease, Oroya fever, or trench fever caused by *Bartonella henselae*, *Bartonella bacilliformis*, and *Bartonella quintana*, respectively. Mortality in humans is generally low; however, the immune status of the patient, the specific *Bartonella* species involved, and the accuracy and timeliness of the treatment are critical factors in preventing fatal outcomes [20]. Although the majority of infections caused in humans are attributed to *Bartonella* species that are not directly associated with rodents (*B. henselae*, *B. quintana*, and *B. bacilliformis*), several rodent-associated *Bartonella* species (*B. elizabethae*, *B. grahamii*, *B. rochalimae*, *B. tamiae*, *B. tribocorum*, *B. vinsonii arupensis*, and *B. washoensis*) compromise human health as they cause clinical manifestations including endocarditis, neuronitis, splenomegaly, fever, and myalgia [7, 20].

In Mexico, the order Rodentia is the most species-rich order of mammals, with 243 species representing eight families. Approximately 112 species have been recorded in north-western Mexico, including the states of Baja California, Sonora, and Chihuahua [21]. The presence of *Bartonella* DNA has been recently recorded in at least 12 rodent species corresponding to seven genera (*Neotoma*, *Onychomys*, *Peromyscus*, *Chaetodipus*, *Dipodomys*, *Cynomys*, and *Xerospermophilus*) in the state of Chihuahua [12]. Despite the scarce research in the country, there is an evidence of zoonotic species in rodents and their fleas in north-western Mexico [9, 12, 22]. It is necessary to continue the surveillance of the bacteria in the north-western region, given that north-western Mexico has a high richness of rodents, and there is evidence that some zoonotic species of *Bartonella* are circulating in rodents and fleas in Chihuahua. Therefore, this study aims to show the prevalence and genetic diversity of *Bartonella* in wild rodents in two north-western Mexican states, Chihuahua and Baja California.

## Materials and methods

### Study area, rodent capture, and blood collection

During 2019 and 2021, rodents were captured in Baja California (32°7′13.59″N, 115°15′4.61″W) and Chihuahua (30°39′8.50″N, 108°31′23.76″W). In both states, agricultural activities are practiced, such as intensive and extensive cattle raising and agriculture, which includes different crop types such as onions, wheat, cotton, and chilli.

Rodent trapping was conducted over two seasons by placing grids of 7×7 Sherman traps (7.6 cm × 8.9cm × 22.9 cm; H.B. Sherman Traps, Inc., Tallahassee, FL). Each grid was spaced at least ≥500m apart. Traps were baited with a mixture of oats and vanilla extract and opened for three consecutive nights. In total, 12 and 18 grids were placed in Baja California and Chihuahua, respectively. The quadrats were distributed in surrounding areas and far from human settlements; most of them were in a mosaic landscape dominated by mesquite shrublands, grassland, and croplands vegetation; and others were placed in oak forest areas.

Once the rodents were captured, they were weighed and sexed, and morphometric measurements were taken for identification using a mammal identification guide [23]. Subsequently, blood samples were obtained from the retro-orbital plexus, placed in cryovials with EDTA, and stored at −70°C. After handling, each rodent was released at the capture site.

### Molecular analysis

DNA extraction from rodent blood samples was performed using the DNeasy Blood and Tissue kit (Qiagen®), following the supplier’s recommendations. Once the extraction was performed, polymerase chain reaction (PCR) was continued by amplifying 767 bp of the *gltA* gene using the following primers: CS443f: 5′ GCTATGTCTGCATTCTCTCTCTCTCTCTATCA 71 3′ and CS1210r: 5′ GATCYTCAATCATTTCTTTCCA 3′ [24]. Amplification consisted of a 25 μl final volume mix containing 12.5 μl Top Taq® Master Mix, 5 μl nuclease-free water, 2.5 μl CoralLoad buffer, 2.5 μl DNA, and 1.25 μl (10 μM) of each primer using the following parameters: initial denaturation (2 min 94°C), followed by 45 cycles at 94°C for 30 s, 48°C for 1 min, and 72°C for 1 min, and a final extension of 72°C for 7 min. The PCR products were visualized for amplicons of the expected size by electrophoresis in a 1% agarose gel with ethidium bromide staining. Some amplicons were purified from an agarose gel (1.5%) with the EZ-10 Spin Column DNA Gel Extraction kit (Bio Basic). Subsequently, purification of the remaining PCR products, and sequencing which was performed in both directions, was carried out in Korea by Macrogen. The phylogenetic relationship of our sequences and the reference sequences obtained from GenBank were aligned using MUSCLE in the MEGA 11 program [25]. Using the same program, the phylogenetic tree was reconstructed by maximum likelihood by Tamura 3-parameter method and a bootstrap calculation with 1000 replicates. The visualization and modification of the phylogenetic tree style were carried out at Fig Tree version 1.4.4. The novel sequences were submitted to GenBank (PQ655038-PQ655044).

## Results and discussion

### *Bartonella* prevalence

A total of 586 rodents belonging to four families (Cricetidae, Heteromyidae, Muridae, and Sciuridae), 12 genera, and 28 species were captured (Table 1). PCR was performed on 408 rodents (Chihuahua=285 and Baja California= 123) obtaining an overall prevalence of *Bartonella* spp. of 39.71%. The highest prevalence was found in the state of Chihuahua with 42.80%, followed by Baja California with a prevalence of 32.52% (Table 1). Previously, a study conducted in 2014 in the same area in Chihuahua reported a higher prevalence in rodents (50.01%) [12], and later in 2016, a prevalence of 40.4% was found in wild rodent fleas [9]. To our knowledge, our study is the first to report *Bartonella* DNA in wild rodents in Baja California; this provides new information in this state, and together with previous studies conducted in Chihuahua and Sonora with rodents and/or their fleas, we confirm the circulation of this bacterium in the three north-western states of Mexico (Baja California, Sonora, and Chihuahua) that border the United States [9, 12, 22].

**Table 1.** Prevalence of *Bartonella* spp. in wild rodents from Chihuahua and Baja California

	Chihuahua					Baja California				
Rodent species	C	A	(+)	(–)	%	C	A	(+)	(–)	%
*Baiomys taylori*	2	–	–	–	–	0	–	–	–	–
*Chaetodipus baileyi*	0	–	–	–	–	1	1	1	0	100
*Chaetodipus eremicus*	2	–	–	–	–	0	–	–	–	–
*Chaetodipus hispidus*	4	2	1	1	50	0	–	–	–	–
*Chaetodipus intermedius*	30	18	8	10	44.44	0	–	–	–	–
*Chaetodipus penicillatus*	6	6	1	5	16.66	65	55	19	36	34.54
*Chaetodipus* sp.	3	1	0	1	0	0	–	–	–	–
*Chaetodipus spinatus*	0	–	–	–	–	7	5	1	4	20
*Dipodomys merriami*	67	44	19	25	43.18	7	2	1	1	50
*Dipodomys ordii*	8	5	2	3	40	0	–	–	–	–
*Dipodomys spectabilis*	5	2	1	1	50	0	–	–	–	–
*Mus musculus*	14	8	1	7	12.5	16	13	4	9	30.76
*Neotoma albigula*	25	18	13	5	72.22	2	2	1	1	50
*Neotoma mexicana*	1	1	1	0	100	0	–	–	–	–
*Onychomys arenicola*	48	32	14	18	43.75	0	–	–	–	–
*Onychomys leucogaster*	14	11	6	5	54.54	0	–	–	–	–
*Perognathus flavus*	21	8	2	6	25	0	–	–	–	–
*Peromyscus boylii*	9	6	6	0	100	0	–	–	–	–
*Peromyscus eremicus*	31	24	13	11	54.16	14	12	6	6	50
*Peromyscus fraterculus*	0	–	–	–	–	3	3	1	2	33.33
*Peromyscus leucopus*	22	20	9	11	45	0	–	–	–	–
*Peromyscus maniculatus*	79	56	23	33	41.07	33	26	6	20	23.07
*Peromyscus* sp.	1	–	–	–	–	0	–	–	–	–
*Rattus rattus*	0	–	–	–	–	2	2	0	2	0
*Reithrodontomys fulvescens*	27	18	1	17	5.55	0	–	–	–	–
*Reithrodontomys megalotis*	0	–	–	–	–	3	1	0	1	0
*Sigmodon hispidus*	7	2	0	2	0	0	–	–	–	–
*Sigmodon ochrognathus*	1	1	0	1	0	0	–	–	–	–
*Xerospermophilus spilosoma*	3	2	1	1	50	0	–	–	–	–
*Xerospermophilus tereticaudus*	0	–	–	–	–	3	1	0	1	0
Total	430	285	122	163	42.80	156	123	40	83	32.52

*Note:* Capital letters represent total rodents captured (C) and abundance of rodents considered for PCR (A)

We found *Bartonella* spp. in several rodent species (Table 1). In Chihuahua, we found 18 rodent species infected with *Bartonella* of which seven are new records (*Chaetodipus intermedius*, *Mus musculus*, *Neotoma mexicana*, *Perognathus flavus*, *Peromyscus boylii*, *Peromyscus eremicus*, and *Reithrodontomys fulvescens*). The species with the highest prevalence in Chihuahua were *Neotoma albigula* (72.22%), *N. mexicana* (100%), and *P. boylii* (100%) (Table 1). The high prevalence found in *N. albigula* may be common, as previous studies in the same state and in New Mexico, United States and adjacent to Chihuahua, recorded a high prevalence in this rodent species (75%–100%) [12, 19]. The prevalence we found in *N. mexicana* and *P. boylii* is higher than that reported for these species in the United States, 38.7% and 33.3%, respectively [26, 27]. In Baja California, *Bartonella* DNA was found in nine rodent species, with the highest prevalence being *Chaetodipus baileyi* (100%), followed by *D. merriami* and *N. albigula* (each 50%). The prevalence found in *D. merriami* and *N. albigula* species has been previously reported as moderate to high [12, 19, 28], but caution should be taken with the high prevalence we found in *C. baileyi*, since the only captured individual was positive. Therefore, further studies to confirm this observation is necessary.

### Phylogenetic analysis

**Figure 1.**
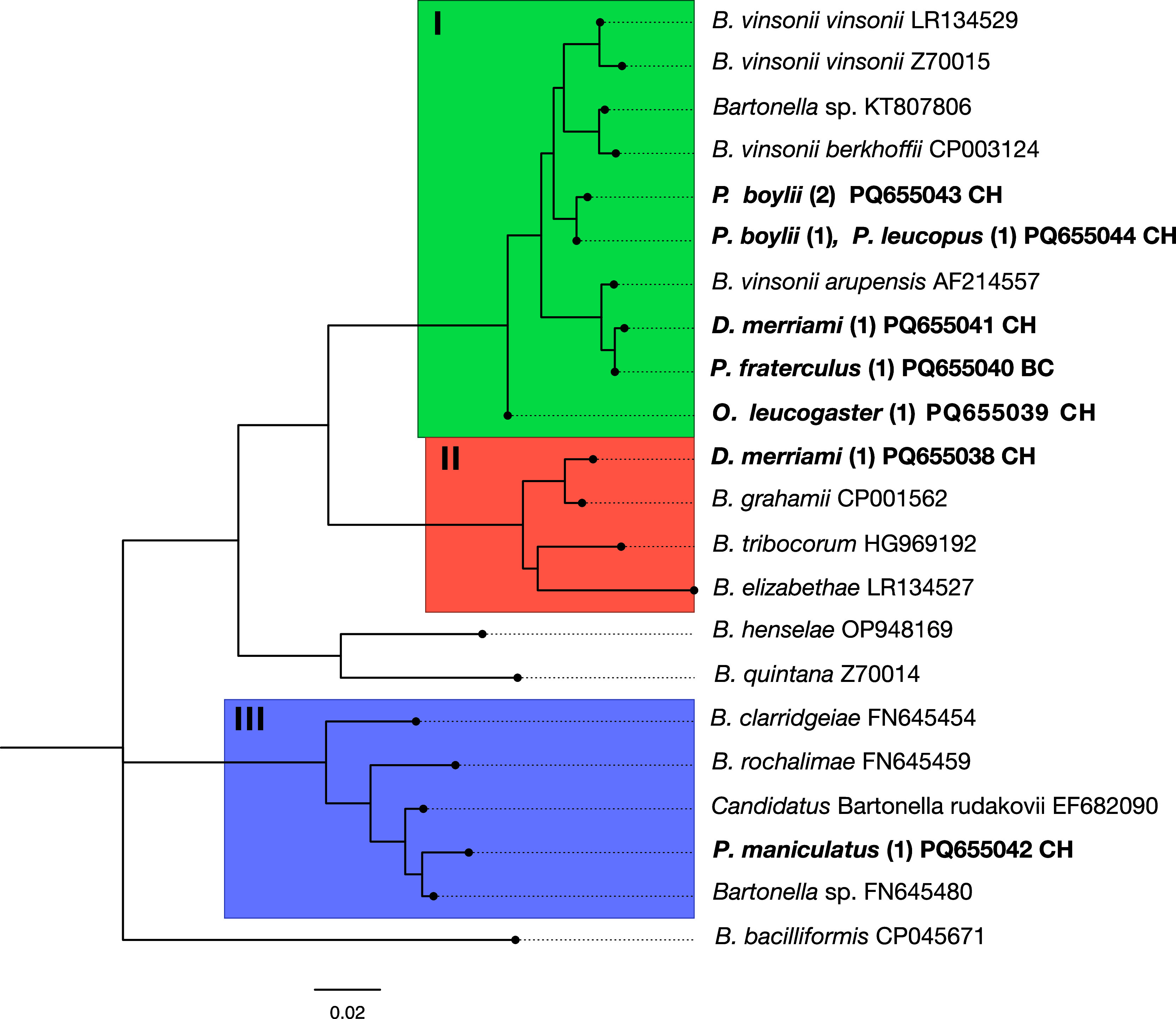
Phylogenetic relationship of *Bartonella* genotypes based on partial sequences of *gltA* gene detected in rodents captured in Baja California (BC) and Chihuahua (CH), Mexico. Each genetic variant is indicated in boldface with its accession number, capital letters show the state where the rodents were captured (CH and BC), and numbers in parentheses are the number of sequences obtained from blood. The clades of interest are represented by a rectangle of different colour and by roman numerals (I–III). The phylogenetic tree was constructed by the maximum likelihood method by Tamura 3-parameter and a bootstrap calculation with 1000 replicates.

A total of 26 positive PCR products were sequenced, with both forward and reverse sequencing performed on each. Of these, only 18 consensus sequences were successfully recovered. Basic Local Assignment Search Tool (BLAST) analysis yielded similarity and query cover values for eight sequences. These sequences were subsequently deposited in GenBank. Notably, some sequences were identical and thus were deposited with the same accession number (PQ655043, PQ655044), as indicated in the supplementary material table. Seven genetic variants were determined and grouped into three clades (Figure 1). In the first clade (I), there were five variants belonging to the rodents *P. boylii* (3), *P. leucopus* (1), *D. merriami* (1), *Peromyscus fraterculus* (1), and *O. leucogaster* (1) (accession numbers: PQ655039-PQ655041, PQ655043, and PQ655044). These variants were associated with the *B. vinsonii* group that include subspecies that have been categorized as pathogenic (*B. vinsonii arupensis* and *B. vinsonii berkhoffii*) [7, 29]. Particularly, the variants belonging to a *D. merriami* (PQ655041, 595 bp) captured in Chihuahua and a *P. fraterculus* (PQ655040, 463 bp) from Baja California were grouped with *B. vinsonii arupensis* (AF214557) with 98.90% and 99.14% similarity.

Previously, in the state of Chihuahua, the presence of *B. vinsonii* subsp. *arupensis* has been reported in rodents such as *N. albigula*, *P. maniculatus*, and *P. leucopus*; however, there was no record of this bacterium in *D. merriami* [12]. In addition, our study adds another rodent species in Chihuahua as carrier of *Bartonella* (*P. boylii*). On the other hand, this is the first time that *B. vinsonii* subsp. *arupensis* is reported in a *P. fraterculus* rodent from Baja California, Mexico.

The second clade (II) consisted of a variant obtained from a *D. merriami* captured in Chihuahua (PQ655038, 737 bp) that had a 98.78% similarity to *B. grahamii* (CP001562), and other species that also parasitize rodents (*B. elizabethae* and *B. tribocorum*). A study conducted in the same state with wild rodents did not report the presence of *B. grahamii* [12]; however, an investigation conducted on rodent fleas reported the presence of this bacterium in *Meringis altipecten*, *Meringis arachis*, and *Meringis parkeri* fleas collected on *D. merriami* [9]. This suggests that *B. grahamii* was already circulating among rodents in the region, despite not having been detected in them until now. *B. grahamii* was recognized of medical importance from its isolation in ocular fluids of a patient, and this *Bartonella* species has been associated with several genera of the order Rodentia (*Apodemus*, *Dryomys*, *Microtus*, *Mus*, and *Myodes*) [30, 31].

The third clade (III) consisted of zoonotic *Bartonella* species mainly associated with carnivores (*B. rochalimae* and *B. clarridgeiae*); however, one of our variants obtained from a *P. maniculatus* collected in Chihuahua (PQ655042, 748 bp) had a similarity of 98.26% with *Bartonella* sp. (FN645480) and 97.91% with *Candidatus* Bartonella rudakovii (EF682090). The later was originally detected in voles from Siberia, and recently using genes such as *gltA* and *rpoB*, it has been recorded in rodents *Myodes glareolus*, *Microtus oeconomus*, and *Sciurus vulgaris* rodents in Switzerland, Lithuania, and Czech Republic [32–34]. In general, information on *Candidatus* B. rudakovii worldwide is scarce, and so far, it has not been recognized as zoonotic [35]. This is the first time that a sequence associated with this putative species of *Bartonella* has been obtained in Mexico, although it has not been recognized as zoonotic, it is found within the clade that integrates species such as *B. clarridgeiae* and *B. rochalimae* which compromise human health.

It is important to note that certain rodent species can adapt to environments near human settlements, where they exploit available resources for their survival. This proximity can increase the likelihood of rodent-human interactions, potentially facilitating the transmission of bacteria or other infectious agents. In our study, some of the cricetid and heteromyid species (*N. albigula*, *N. mexicana*, *P. boylii*, *C. baileyi*, *D. merriami*, and *P. fraterculus*) presented high prevalence and/or zoonotic species of *Bartonella*; these rodents are usually found far from human settlements, as they are distributed in landscapes composed of forests, shrublands, deserts, and grasslands, so the risk of transmission of *Bartonella* bacteria to humans could be low [23]. However, some rodent species found in our study, such as *N. albigula* and *N. mexicana*, may occasionally be found in abandoned buildings [36, 37]. Furthermore, it should be considered that some human activities such as agricultural and livestock production, animal trade, deforestation, travel, and tourism, among others, imply the entry of humans into areas inhabited by wild animals, which increases the probability of contact and, in turn, the risk of transmission of *Bartonella* or other infectious agents [38].

Our study, in conjunction with existing research, indicates that *Bartonella* is a persistent agent in wild rodents in north-western Mexico. The identification of certain sequences that correspond to zoonotic species underscores the necessity for preventive measures to avert the dissemination and occurrence of cases in humans. *Bartonella* species identification was carried out with the *gltA* gene, which is a reliable and widely used gene; however, for further studies, we recommend the use of multiple genes to discern between *Bartonella* species [17, 39]. Additionally, it is recommended that further research be conducted on this bacterium in Baja California to gain a deeper understanding of its prevalence and diversity within the state.

## Supporting information

Fernández-González et al. supplementary material Fernández-González et al. supplementary material

## Data Availability

The genetic variants are all available at the NCBI repository as described in materials and methods under accession numbers (PQ655038-PQ655044).

## Supplementary material

For supplementary material accompanying this paper visit http://doi.org/10.1017/S0950268825000238. click here to view supplementary material
