# Supplementary material for: Bartonella tracing in wild rodents in northwestern Mexico
Source: Epidemiol Infect. 2025 Feb 28;153:e42. doi: 10.1017/S0950268825000238 (PMC11920920; doi:10.1017/S0950268825000238)
Supplement: Fernández-González et al. supplementary material [file S0950268825000238sup001.docx]

**Sequences and rodent data**

| **Accession number of genetic variants** | **Clade in phylogenetic analysis** | **Rodent species** | **State of capture** | **Date of rodent capture** |
| --- | --- | --- | --- | --- |
| PQ655038 | II | *Dipodomys merriami* | Chihuahua | 24-Feb-2020 |
| PQ655039 | I | *Onychomys leucogaster* | Chihuahua | 29-Feb-2020 |
| PQ655040 | I | *Peromyscus fraterculus* | Baja California | 19-Sep-2021 |
| PQ655041 | I | *Dipodomys merriami* | Chihuahua | 29-Feb-2020 |
| PQ655042 | III | *Peromyscus maniculatus* | Chihuahua | 01-Mar-2020 |
| PQ655043 | I | *Peromyscus boylii* | Chihuahua | 03-Mar-2020 |
| PQ655044 | I | *Peromyscus boylii and Peromyscus leucopus* | Chihuahua | 03-Mar-2020 |
